# Denitrification Activity of a Remarkably Diverse Fen Denitrifier Community in Finnish Lapland Is N-Oxide Limited

**DOI:** 10.1371/journal.pone.0123123

**Published:** 2015-04-10

**Authors:** Katharina Palmer, Marcus A. Horn

**Affiliations:** Department of Ecological Microbiology, University of Bayreuth, Bayreuth, Germany; Netherlands Institute of Ecology (NIOO/KNAW), NETHERLANDS

## Abstract

Peatlands cover more than 30% of the Finnish land area and impact N_2_O fluxes. Denitrifiers release N_2_O as an intermediate or end product. *In situ* N_2_O emissions of a near pH neutral pristine fen soil in Finnish Lapland were marginal during gas chamber measurements. However, nitrate and ammonium fertilization significantly stimulated *in situ* N_2_O emissions. Stimulation with nitrate was stronger than with ammonium. N_2_O was produced and subsequently consumed in gas chambers. In unsupplemented anoxic microcosms, fen soil produced N_2_O only when acetylene was added to block nitrous oxide reductase, suggesting complete denitrification. Nitrate and nitrite stimulated denitrification in fen soil, and maximal reaction velocities (*v_max_*) of nitrate or nitrite dependent denitrification where 18 and 52 nmol N_2_O h^-1^ g_DW_^-1^, respectively. N_2_O was below 30% of total produced N gases in fen soil when concentrations of nitrate and nitrite were <500 μM. v_max_ for N_2_O consumption was up to 36 nmol N_2_O h^-1^ g_DW_^-1^. Denitrifier diversity was assessed by analyses of *narG*, *nirK/nirS*, and *nosZ* (encoding nitrate-, nitrite-, and nitrous oxide reductases, respectively) by barcoded amplicon pyrosequencing. Analyses of ~14,000 quality filtered sequences indicated up to 25 species-level operational taxonomic units (OTUs), and up to 359 OTUs at 97% sequence similarity, suggesting diverse denitrifiers. Phylogenetic analyses revealed clusters distantly related to publicly available sequences, suggesting hitherto unknown denitrifiers. Representatives of species-level OTUs were affiliated with sequences of unknown soil bacteria and *Actinobacterial*, *Alpha-*, *Beta-*, *Gamma-*, and *Delta-Proteobacterial* sequences. Comparison of the 4 gene markers at 97% similarity indicated a higher diversity of *narG* than for the other gene markers based on Shannon indices and observed number of OTUs. The collective data indicate (i) a high denitrification and N_2_O consumption potential, and (ii) a highly diverse, nitrate limited denitrifier community associated with potential N_2_O fluxes in a pH-neutral fen soil.

## Introduction

Northern peatlands are important players in the global carbon and nitrogen cycles, and store more than 30% of soil carbon and nitrogen even though they cover only about 3% of the terrestrial surface [1]. Greenhouse gases such as methane (CH_4_) and nitrous oxide (N_2_O) are produced in and released from northern peatlands soils [2]. High latitude peatlands have been intensively studied with respect to their capacity to emit CH_4_ due to the large amount of stored carbon in peat soils (e.g., [3–5]). N_2_O has a high global warming potential (approximately 300 times higher than CO_2_), is a major ozone-depleting substance, and 6% of the greenhouse effect is attributed to N_2_O [6–8]. Stored organic N in certain peatlands fuels N_2_O release via coupling of ammonification, ammonia oxidation, and denitrification [9]. Thus, potential N_2_O emissions from northern peatlands are of major interest. Northern peatlands are very diverse ecosystems, including many types of pristine and managed soils. Many studies investigating N_2_O emissions from peatlands have focused on N_2_O emissions from managed peatlands, and only recently N_2_O fluxes from pristine northern peat soils have been investigated [10–15]. Pristine northern fens include significant net sources of N_2_O even though emission rates are generally low [10, 13]. Negative N_2_O fluxes suggest that peatlands can act as temporary sinks for N_2_O [2, 14, 16, 17]. Understanding of the mechanisms and regulation of N_2_O fluxes in such systems is still incomplete.

N_2_O in soils is generally produced during nitrification, denitrification, or chemical processes [18, 19]. Denitrification is considered to be the main source of N_2_O in water-saturated soils including peatlands [19, 14]. During denitrification, nitrate or nitrite are sequentially reduced via nitric oxide (NO) and N_2_O to dinitrogen (N_2_) [20]. The reductions are catalyzed by a set of oxidoreductases, namely nitrate reductases (encoded by *narG* and *napA*), nitrite reductases (encoded by *nirK* and *nirS*), NO reductases (endoded by *norBC*), and N_2_O reductases (encoded by *nosZ*) [20]. N_2_O or N_2_ can be released into the atmosphere. The ratio of N_2_O to N_2_ is determined by *in situ* parameters such as pH, temperature, as well as nitrate/nitrite and electron donor availability [21].

High latitude peatlands are likely to be strongly affected by increasing temperatures due to climate change [3, 4, 9, 22]. Global warming might reduce the water table in northern peatlands and influence the amount of N_2_O released from the soil [9]. A constantly lowered water table increases N_2_O fluxes from nutrient rich peat, whereas fluxes from nutrient-poor peat remain largely unaffected [12]. Dissimilar denitrifier communities are related with dissimilar N_2_O fluxes [15]. Detailed knowledge about the microbial catalysts involved in N_2_O turnover in northern peatlands is scarce. Thus, the aim of this study was to assess denitrification in a pH neutral pristine fen. The main objectives were to (i) assess *in situ* N_2_O emissions of a pH-neutral fen soil, (ii) determine depth-related N_2_O production and consumption capacities of fen soil, and (iii) link differences in the denitrifier community composition to physiological differences of the denitrification in the peat soil over two depths.

## Material and Methods

### Sampling site and soil parameters

Puukkosuo fen is located in northeastern Finland (66°22'38''N, 29°18'28''E) at an elevation of 220 m above sea level. The mean annual air temperature is (-0.43±0.09)°C, and mean annual precipitation approximates (772±12) mm (average of years 1966 to 2011, measured at Oulanka research station). The fen is meso-eutrophic and water saturated. Vegetation consists mainly of mosses (*Sphagnum* spp.) and grasses (e.g., *Carex* spp.). Four replicate soil cores from layers 0 to 20 cm and 20 cm to 40 cm were taken on July 28th 2010. Soil temperatures on the day of sampling were 17.2°C in surface soil and 15.1°C in deeper soil layers (below 15 cm). Samples were transported on ice to the laboratory and stored at 4°C for microcosm analyses or at -80°C for nucleic acid extractions. Microcosm experiments were conducted within 2 weeks after sampling. Nitrate, nitrite and ammonium concentrations, soil pH, soil moisture content, total carbon (TC), dissolved organic carbon (DOC) and total nitrogen (TN) were determined from pooled soil samples as described previously [23]. Permission to access and sample Puukkosuo fen was granted by Metsähallitus (www.metsa.fi) on 12th of July 2010.

### Assessment of *in situ* gas emissions

*In situ* gas emissions of unfertilized soil and soil supplemented with either nitrate or ammonium were determined in closed poly(methyl methacrylate) (PMMA) chambers. Chambers were placed onto the soil surface and surrounded by metal collars, which had been inserted into the soil for a few centimeters to ensure that the chambers were gas tight. The transition between the plexiglas chamber and the metal ring was sealed with a rubber band to avoid exchange of gases from the chamber with the surrounding air. Before the installation of the gas chambers, 2l of fen pore water with 20 mM of added nitrate or ammonium was applied homogeniously onto the soil surface in 4 replicate treatments each and unsupplemented controls received pure fen pore water. Gas samples (5 ml per sampling timepoint) were taken from gas outlets and injected into gas tight evacuated containers (Exetainer, Labco Limited, High Wycombe, UK) at the start of the experiment, after 0.5, 1 and 3 hours.

### Assessment of denitrification potentials in soil microcosms

Denitrification potentials of pH-neutral fen soil (0 to 20 cm and 20 to 40 cm) were assessed in unsupplemented and nitrate-, nitrite- or N_2_O-supplemented anoxic microcosms as described earlier [14, 15, 23]. Supplemental nitrate and nitrite ranged from 0 to 1000 μM, while supplemental N_2_O ranged from 0 to 4 μM. Acetylene blockage was used to distinguish between total N_2_O production and total denitrification as described earlier [15, 24]. Incubations were conducted at 20°C in the dark. N_2_O production rates and apparent kinetic parameters [Michaelis-Menten constants (*K*_*M*_) and maximum reaction velocitites (*v*_*max*_)] were determined as described [14]. Michaelis-Menten regressions obtained for different incubation conditions were compared using the “extra sum of squares” principle to test for significant differences between the regressions [25]. Obtained values for *K*_*M*_ and *v*_*max*_ were compared by t-tests.

### Molecular characterisation of fen denitrifier communities

Nucleic acids were extracted from homogenized pooled fen soil of both soil layers as previously described using a bead-beating protocol [15, 26]. DNA yields were 4 to 12 μg DNA per gram (fresh weight) of soil. A_260_/A_230_ values approximating 0.94 to 1.56 indicated DNA with moderate to low humic acid content. The structural genes *narG*, *nirK*, *nirS*, and *nosZ* were amplified using the primer pairs narG1960f (TAY GTS GGS CAR GAR AA)/narG2650r (TTY TCR TAC CAB GTB GC) [27], F1aCu (ATC ATG GTS CTG CCG CG)/R3Cu (GCC TCG ATC AGR TTG TGG TT) [28], cd3aF (GTS AAC GTS AAG GAR ACS GG)/R3cd (GAS TTC GGR TGS GTC TTG A) [28], and nosZF (CGC TGT TCI TCG ACA GYC AG)/nosZR (ATG TGC AKI GCR TGG CAG AA) [29], respectively, and subjected to barcoded pyrosequencing as previously described [15, 23]. Barcodes used to identify sequences after pyrosequencing were ACTGCG and AGTATG for 0 to 20 cm and 20 to 40 cm fen soil, respectively. Pyrosequencing was performed at the Göttingen Genomics Laboratory using the Roche GS-FLX 454/Titanium technology as previously described [15, 23]. Pyrosequencing and PCR errors of the obtained reads were corrected using the AmpliconNoise pipeline [30] and sequences were clustered at species-level (i.e., for *narG*, *nirK*, *nirS*, and *nosZ*, respectively), and 97% threshold similarities using Qiime as previously described [23, 31]. Species-level threshold similarities were determined from pairwise comparisons of 16S rRNA gene similarities and structural gene similiarities of cultured denitrifiers [32]. Such OTUs indicate a minimal estimate of species-level diversity, i.e., is likely to underestimate “real” species-level diversity. Phylogenetic trees with cluster representatives were constructed in MEGA 5.0 [33]. Alpha- and beta-diversity measures were calculated in Qiime from rarified OTU tables as described [23, 34] to allow statistical comparison of the structural gene diversity from both soil layers. Rarified OTU tables were generated in Qiime by randomly subsampling original OTU tables 100 times at depth of 1000, 1500, 2500, and 500 sequences for *narG*, *nirK*, *nirS*, and *nosZ*, respectively. OTU representative sequences of *narG*, *nirK*, *nirS*, and *nosZ* were deposited at EMBL under accession numbers HE995549 to HE995577. Complete sequence data sets were deposited deposited in the European Nucleotide Archive (ENA) under the study accession number ERP008864.

Quantitative kinetic real-time PCRs (qPCRs) were performed in 6 technical replicates as described [15]. Obtained gene copy numbers were corrected for inhibition with inhibition factors ranging from 0.5–0.6, 0.3–0.4, 0.5–1.0, 0.9–1.0, and 0.9–1.0 for *narG*, *nirK*, *nirS*, *nosZ*, and 16S rRNA genes, respectively [35]. Normal distribution of the data was verified by Kolmogorov-Smirnov as well as Shapiro-Wilk tests. Copy numbers of *narG*, *nirK*, *nirS*, and *nosZ* in 0 to 20 cm and 20 to 40 cm soil were statistically evaluated using Student’s t-test (based on the 6 replicates for each gene).

## Results

### Soil parameters

Soil moisture content of Puukkosuo fen soil was 90% in both soil layers (Table 1). Soil pH in water was 6.8 and 6.9 in 0 to 20 cm and 20 to 40 cm fen soil, respectively. Nitrate was below the detection limit of 5.8 μg g_DW_^-1^ (Table 1). Values for carbon and nitrogen contents appeared to be marginally higher in 20 to 40 cm than in 0 to 20 cm fen soil, but C/N ratios and DOC concentrations were similar in both soil layers (Table 1).

**Table 1:** Soil parameters of Puukkosuo fen.

Soil layer (cm)	pH	Moisture	NO_3_^-^		NO_2_^-^		NH_4_^+^		Total C ^1^	DOC ^2^	Total N^3^	C/N ^4^
		content (%)	(μM)	(μg g_DW_^-1^)	μM	(μg g_DW_^-1^)	(μM)	(μg g_DW_^-1^)	(g kg_DW_^-1^)	(mg l^-1^)	(g kg_DW_^-1^)	
0 to 20	6.8	90	14.4	7.7	< 48.5	< 19.2	77.2	11.9	434	63.2	29	15
20 to 40	6.9	90	< 10.7	< 5.8	< 48.2	< 19.2	37.7	5.9	492	65.1	35	14
Pore water		n.a. ^5^	< 2.5	n.a. ^5^	< 10.9	n.a. ^5^	< 1.4	n.a. ^5^	n.a. ^5^	8,8	n.a. ^5^	n.a. ^5^

^1^ Total carbon

^2^ Dissolved organic carbon (per l porewater)

^3^ Total nitrogen

^4^ Carbon to nitrogen ratio

^5^ Not applicable

### *In situ* gas emissions of fen soil

**Fig 1:**
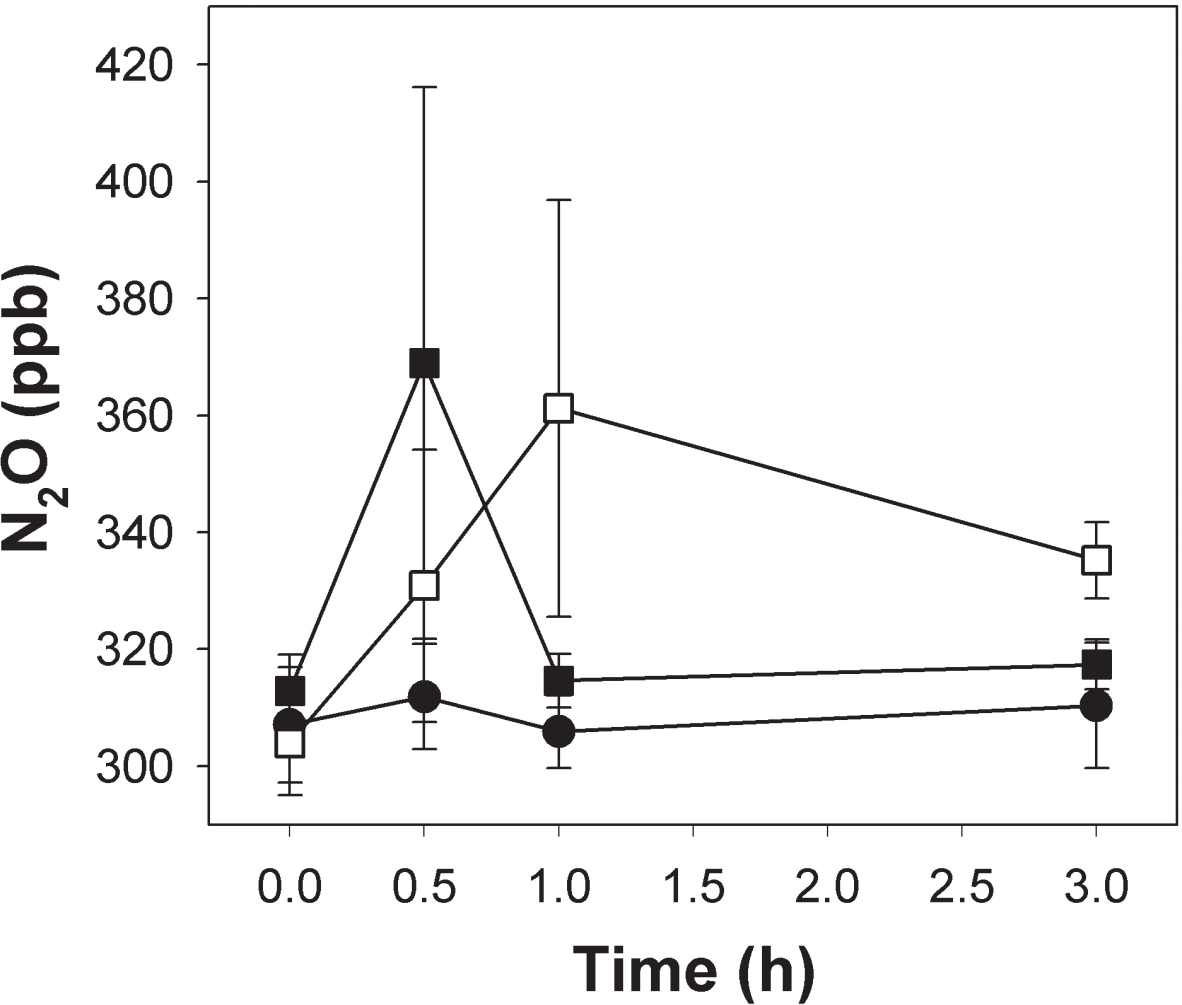
Effect of *in situ* nitrate and ammonium fertilization on N_2_O accumulation in closed chambers from pH-neutral fen soil. Mean values and standard errors of 4 replicates are displayed. Closed circles represent unfertilized controls, closed and open squares represent soil fertilized with 20 mM NaNO_3_ and 20 mM NH_4_Cl, respectively.

During gas chamber measurements, only minor amounts of N_2_O accumulated in gas chambers placed on unsupplemented fen soil on average (Fig 1). Increases of about 10 ppb in N_2_O mixing ratio were observed in two of the four replicate gas chambers, while decreases in N_2_O mixing ratio were observed in the other two replicate gas chambers (-1 to -17 ppb decrease in mixing ratio). Nitrate-addition initially lead to accumulation of N_2_O in the gas chambers. However, this accumulation of N_2_O was restricted to the first 30 minutes after nitrate-addition, and initially accumulated N_2_O was subsequently consumed after 30 minutes (Fig 1). Ammonium likewise led to accumulation of N_2_O in the gas chambers, however this initial accumulation of N_2_O was slower than after nitrate-addition (Fig 1). Moreover, initially accumulated N_2_O was subsequently consumed after the first hour.

### Denitrification potentials in fen soil microcosms

**Fig 2:**
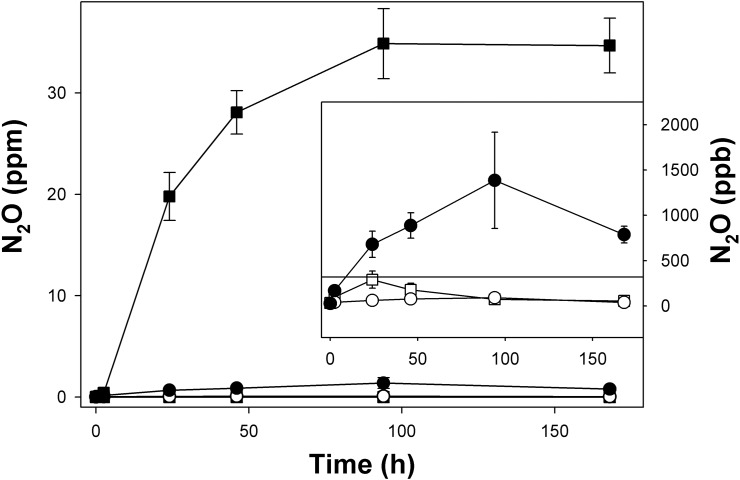
N_2_O production and consumption in unsupplemented anoxic fen soil microcosms. Squares and circles represent 0 to 20 cm and 20 to 40 cm fen soil, respectively. Open and closed symbols represent microcosms without and with acetylene addition, respectively. Mean values and standard errors of three replicates are displayed. The inset represents an enlargement of the lower N_2_O mixing ratios to allow better visualization of N_2_O production and consumption in 20 to 40 cm fen soil and microcosms without acetylene addition. The horizontal line indicates the atmospheric N_2_O mixing ratio (319 ppb).

In anoxic microcosms, unsupplemented fen soil from both soil layers produced only minor amounts of N_2_O in the absence of acetylene, and initially produced N_2_O was subsequently consumed (Fig 2). However, N_2_O production was significantly higher in anoxic microcosms when N_2_O-reductase was blocked by acetylene (Fig 2). N_2_O mixing ratios increased from 0.04 ± 0.004 to about 35 ± 3.5 ppm within the first 94 hours in acetylene-amended microcosms with 0 to 20 cm fen soil, and the concentration of N_2_O plateaued out after the first 94 hours (Fig 2), indicating that endogenous nitrate had been consumed. The increase in N_2_O mixing ratio was significantly lower in microcosms with 20 to 40 cm fen soil than in those with 0 to 20 cm fen soil (Fig 2). N_2_O mixing ratios increased from 0.03 ± 0.001 to 1.4 ± 0.5 ppm within the first 94 hours in acetylene-amended microcosms with 20 to 40 cm fen soil. In 20 to 40 cm fen soil microcosms without acetylene, mixing ratios increased only to 91 ± 21 bbp N_2_O within the first 94 hours. The initially accumulated N_2_O was subsequently consumed within the next 74 hours. Both soil layers displayed the capability to consume subatmospheric concentrations of N_2_O in microcosms without acetylene. 0 to 20 cm fen soil reduced N_2_O from 290 ppb to 55 ppb, while 20 to 40 cm fen soil reduced N_2_O from 91 ppb to 39 ppb (Fig 2).

**Fig 3:**
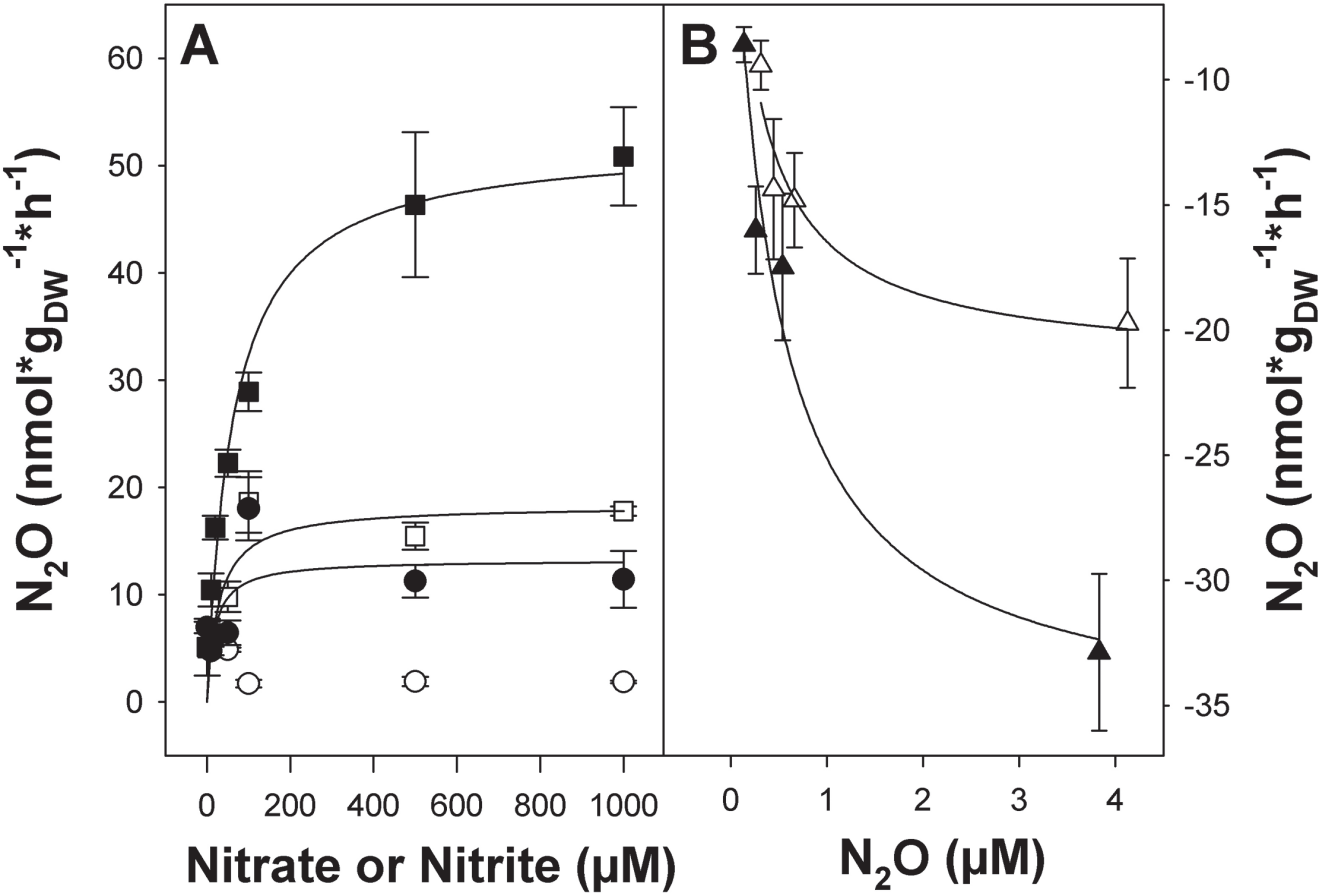
Effect of added nitrate, nitrite (A) and N_2_O (B) on N_2_O production and consumption in anoxic fen soil microcosms. Closed and open symbols represent 0 to 20 cm and 20 to 40 cm fen soil microcosms, respectively. Circles, squares and triangles represent microcosms supplemented with nitrate, nitrite or N_2_O, respectively. Mean values and standard errors of three replicates are displayed. The x-axis displays the amount of supplemented (i.e., additional) nitrate, nitrite or N_2_O. Solid lines indicate Michaelis-Menten curves fitted to the data.

Supplemental nitrate and nitrite stimulated the production of N_2_O without apparent delay in microcosms with fen soil from both soil layers (Fig 3 A and S1 Fig), while N_2_O consumption was stimulated in N_2_O supplemented microcosms (Fig 3 B). Stimulation of N_2_O production with nitrate was smaller than with nitrite, and N_2_O production in nitrate-supplemented microcosms was less than 25% of that in nitrite-supplemented microcosms (Fig 3 A and S1 Fig). In microcosms with fen soil from 0 to 20 cm, N_2_O production in acetylene-amended microcosms was in a similar magnitude for all supplemented nitrate concentrations ≥100 μM. N_2_O production in microcosms with fen soil from 20 to 40 cm was highest when 50 μM nitrate were supplied, and decreased with increasing nitrate concentrations, indicating that denitrifiers in fen soil were saturated at low nitrate concentrations, and were inhibited by higher nitrate concentrations (Fig 3 A). In nitrite-supplemented microcosms, N_2_O production rates increased with increasing nitrite concentrations in both soil layers (Fig 3 A). N_2_O consumption was likewise stimulated by increasing N_2_O concentrations (Fig 3 B). N_2_O production and consumption capacities were higher in 0 to 20 cm fen soil than in 20 to 40 cm fen soil.

The ratio of N_2_O to (N_2_ + N_2_O) was below 30% and 40% for all supplied nitrate concentrations in microcosms with 0 to 20 cm and 20 to 40 cm fen soil, respectively (S2 Fig), indicating that more than half of the N_2_O produced from nitrate was further reduced to N_2_ in fen soil. The ratio of N_2_O to (N_2_ + N_2_O) was below 30% in microcosms with fen soil from 0 to 20 cm when nitrite concentrations were 100 μM or smaller and increased to about 75% for higher nitrite concentrations. In microcosms with 20 to 40 cm fen soil, the ratio of N_2_O to (N_2_ + N_2_O) was between 50% and 100% for all supplied nitrite concentrations (S2 Fig), indicating that N_2_O was a major product of denitrification in that soil layer when nitrite was provided as electron acceptor.

Initial nitrite-dependent N_2_O production rates of fen soil microcosms amended with acetylene followed apparent Michaelis-Menten kinetics, as did nitrate-dependent N_2_O production rates of fen soil microcosms from 0 to 20 cm depth and N_2_O-dependent N_2_O consumption rates in both layers (Fig 3). The Michaelis-Menten kinetics differed significantly between the different treatments and soil layers (*p* ≤ 0.03 for all comparisons). Apparent maximal reaction velocities (*v*_*max*_) were highest for nitrite-dependent N_2_O production, followed by N_2_O-dependent N_2_O consumption rates. *v*_*max*_ was lowest for nitrate-dependent N_2_O production (Table 2). *v*_*max*_ values for nitrate and nitrite dependent N_2_O production, as well as N_2_O-dependent N_2_O consumption were significantly higher in 0–20 cm than 20–40 cm fen soil (*p* < 0.001, and *p* < 0.001, as well as *p* = 0.02, respectively). Apparent Michaelis-Menten constants *K*_*M*_ were about 60 to 140 times lower for N_2_O consumption than for nitrite dependent N_2_O production in 0 to 20 cm fen soil (*p* = 0.003), indicating a high affinity of fen denitrifiers for N_2_O (Table 2).

**Table 2:** Kinetic parameters of denitrification in Puukkosuo fen soil.

								
	Nitrate amended			Nitrite amended			N_2_O amended	
Soil layer (cm)	*K*_*M*_ ^1^	*v*_*max*_ ^1^		*K*_*M*_ ^1^	*v*_*max*_ ^1^		*K*_*M*_ ^1^	*v*_*max*_ ^1^
	(μM)	(nmol h^-1^ g_DW_^-1^)		(μM)	(nmol h^-1^ g_DW_^-1^)		(μM)	(nmol h^-1^ g_DW_^-1^)
0 to 20	28.7 ± 16.8	18.3 ± 2.6		61.8 ± 14.2	52.3 ± 3.3		0.43 ± 0.12	- 36.0 ± 3.5
20 to 40	n.a. ^2^	n.a. ^2^		18.3 ± 21.0	13.3 ± 3.3		0.30 ± 0.09	- 21.4 ± 2.0

^1^ Kinetic parameters (calculated from Fig 3) ± standard error.

^2^ Not applicable.

### Phylogenetic analysis of denitrifiers in high latitude peatlands

Approximately 14 000 denoised quality-filtered sequences of the structural gene markers *narG*, *nirK*, *nirS*, and *nosZ* were utilized in total for further analyses. Forward and reverse reads for *nirK* and *nirS* showed a sufficiently long overlap (amplicon lengths of approximately 470 and 410 bp, respectively) to allow combined assessment of forward and reverse reads per gene for further analyses. Only forward reads of *narG* and *nosZ* were analyzed, as the overlap of forward and reverse reads was not sufficient to allow a combined analysis of forward and reverse reads (amplicon lengths approximately 670 and 700 bp for *narG* and *nosZ*, respectively), and previous studies indicate that results obtained from forward and reverse reads of *narG* and *nosZ* are similar [15, 23]. More than 99% of sequences generated from amplicons of a certain gene specific (i.e., *narG*, *nirK*, *nirS*, *nosZ*) primer set were specific amplicons of the target gene. All library coverages were greater than 99% at species-level DNA sequence dissimilarities of 33%, 17%, 18%, and 20% for *narG*, *nirK*, *nirS*, and *nosZ*, respectively, and varied from 80% to 97% at 3% sequence dissimilarity (Table 3), indicating that the number of sequences generated was sufficient.

**Table 3:** Diversity measures of amplicon pyrosequencing libraries of fen soil from rarified and non-rarified OTU tables of *narG*, *nirK*, *nirS* and *nosZ*.

												
Gene marker	Threshold similarity (%)	Soil depth (cm)	Original non-rarified OTU tables			α-Diversity (based on rarified OTU tables)			β-Diversity (based on rarified OTU tables)						
			No. of sequences	Library coverage (%) ^1^	No. of OTUs observed^2^	No. of OTUs estimated ^3^	*H* ^4^	*E* ^5^	*S*_*S*_ ^*6*^		*BC*_*S*_ ^*7*^		*D*_*UU*_ ^*8*^		*D*_*WU*_ ^*9*^
*narG*	67	0 to 20	1 141	99.7	7	9 ± 0.1 (A)	1.28 ± 0.001 (A)	0.46 ± 0.002 (A)	0.31 ±	0.25 ±		0.40 ±		0.20 ±	
		20 to 40	1 697	99.9	4	4 ± 0.1 (B)	1.20 ± 0.002 (B)	0.65 ± 0.007	0.01	0.001		0.006		0.001	
	97	0 to 20	1 141	79.8	359	814 ± 5 (A)	7.03 ± 0.003 (A)	0.84 ± 0.001 (A)	0.78 ±	0.85 ±		0.71 ±		0.56 ±	
		20 to 40	1 697	93.7	230	312 ± 3 (B)	5.35 ± 0.006 (B)	0.72 ± 0.001 (B)	0.001	0.001		0.001		0.001	
*nirK*	83	0 to 20	1 814	99.7	23	26 ± 0.3 (A)	1.53 ± 0.002 (A)	0.34 ± 0.001 (A)	0.22 ±	0.64 ±		0.41 ±		0.30 ±	
		20 to 40	1 876	99.8	17	19 ± 0.4 (B)	1.41 ± 0.002 (B)	0.35 ± 0.001 (B)	0.003	0.001		0.004		0.001	
	97	0 to 20	1 814	93.8	174	381 ± 5 (A)	3.57 ± 0.003 (A)	0.49 ± 0.001 (A)	0.75 ±	0.93 ±		0.67 ±		0.40 ±	
		20 to 40	1 876	96.1	109	256 ± 5 (B)	2.04 ± 0.004 (B)	0.31 ± 0.001 (B)	0.002	0.001		0.002		0.001	
*nirS*	82	0 to 20	3 146	99.9	22	24 ± 0.3	2.14 ± 0.002 (A)	0.49 ± 0.001 (A)	0.13 ±	0.78 ±		0.19 ±		0.32 ±	
		20 to 40	3 382	99.9	23	24 ± 0.2	2.78 ± 0.001 (B)	0.62 ± 0.001 (B)	0.003	0.001		0.005		0.001	
	97	0 to 20	3 146	93.8	301	667 ± 7 (A)	4.34 ± 0.003 (A)	0.54 ± 0.001 (A)	0.78 ±	0.81 ±		0.68 ±		0.40 ±	
		20 to 40	3 382	96.7	185	368 ± 4 (B)	3.84 ± 0.003 (B)	0.53 ± 0.001 (B)	0.001	0.001		0.001		0.001	
*nosZ*	80	0 to 20	572	100.0	8	8 ± 0.0	1.78 ± 0.003 (A)	0.60 ± 0.001 (A)	0.25 ±	0.30 ±		0.30 ±		0.18 ±	
		20 to 40	530	99.8	8	8 ± 0.0	0.79 ± 0.002 (B)	0.26 ± 0.001 (B)	0.001	0.001		0.001		0.001	
	97	0 to 20	572	86.4	120	292 ± 3 (A)	5.10 ± 0.005 (A)	0.75 ± 0.001 (A)	0.68 ±	0.75 ±		0.56 ±		0.47 ±	
		20 to 40	530	96.4	40	80 ± 2 (B)	2.92 ± 0.002 (B)	0.55 ± 0.001 (B)	0.001	0.001		0.001		0.001	

Original OTU tables were rarified 100 times at sequence depths of 1000, 1500, 2500, and 500 for *narG*, *nirK*, *nirS*, and *nosZ*, respectively. Different letters in parentheses indicate that α-diversity measures of 0 to 20 and 20 to 40 cm depth fen soil differed significantly (Student's *T*-test, *p* < 0.001).

^1^ Percent library coverage *C* = (1—*ns/nt*) * 100 (*ns =* OTUs that occur only once, *nt =* total number of sequences).

^2^ Number of OTUs observed in non-rarified OTU tables ± standard error.

^3^ Chao1 richness estimate of rarified OTUs ± standard error.

^4^ Shannon diversity index of rarified OTUs ± standard error.

^5^ Species Evenness of rarified OTUs ± standard error.

^6^ Sørensen similarity index of rarified OTUs ± standard error.

^7^ Bray Curtis similarity index of rarified OTUs ± standard error.

^8^ Unweighted Unifrac distance of rarified OTUs ± standard error.

^9^ Weighted Unifrac distance of rarified OTUs ± standard error.

**Fig 4:**
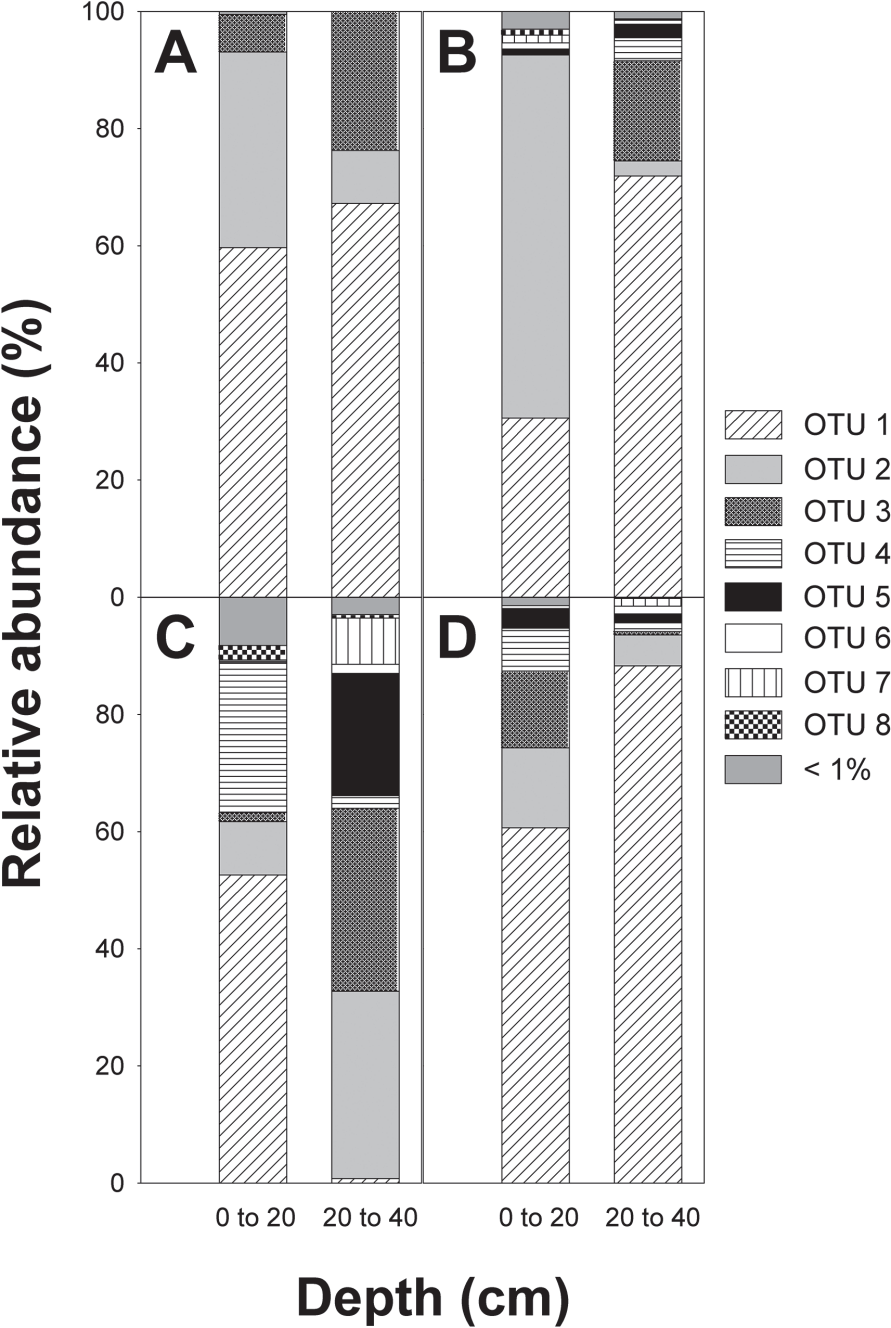
Relative abundances of denitrification associated genes in amplicon libraries of pH-neutral fen soil. OTUs of *narG* forward reads (A), *nirK* (B), *nirS* (C), and *nosZ* forward reads (D) were derived at species-level thresholds of 33%, 17%, 18%, and 20%, respectively.

*narG* sequences were assigned to 7 species-level OTUs in total. 7 and 4 OTUs were detected in 0 to 20 cm and 20 to 40 cm of fen soil, respectively (Table 3). *narG* community composition was similar in both sampled soil layers (Fig 4 A). Three OTUs had a relative abundance greater than 1%. Of those OTUs, OTU 1 dominated *narG* in fen soil (about 60% in both soil layers). About 40% of *narG* belonged to OTUs 2 and 3 (Fig 4 A). OTU 2 was more abundant in 0 to 20 cm than in 20 to 40 cm fen soil (relative abundances of 33% and 9%, respectively), whereas OTU 3 was more abundant in 20 to 40 cm fen soil (23% vs. 6% in 0 to 20 cm fen soil; Fig 4 A). Most of the OTUs were only distantly related to *narG* of cultured organisms or environmental sequences (i.e., sequence dissimilarities of OTU representatives were 10–23%) (Table 4 and S3 Fig). Sequences of OTUs 1, 2, and 3 affiliated with *narG* of *Alphaproteobacteria*, *Actinobaceria*, and *Deinococci*, respectively, more specifically they were related to *narG* of uncultured bacteria and to those of *Oligotropho carboxidovorans*, *Salinispora arenicola*, and *Marinithermus hydrothermalis*, respectively (Table 4 and S3 Fig). Observed *narG* diversity was higher at 97% threshold similarity than at species-level threshold similarity (Table 3). At 97% threshold similarity, 359 and 230 OTUs were detected in 0 to 20 cm and 20 to 40 cm fen soil, respectively (Table 3). Shannon diversity, species evenness indices, and the observed number of OTUs calculated from rarified OTU tables indicated significantly higher diversity in 0–20 cm than 20–40 cm fen soil at 97% and species-level threshold similarity (Table 3). Beta-diversity measures indicated greater differences in community composition at 97% than at 67% threshold similarity (Table 3).

**Table 4:** OTU representatives retrieved from pH-neutral fen soil.

						Relative abundance of OTUs in amplicon libraries (%)	
				
Gene marker	OTU (accesion No.)	Closest relative (accession No.)	Similarity (%) ^1^	Closest cultured relative (accession No.)	Similarity (%) ^1^	0 to 20 cm	Below 20 cm
*narG*	1 (HE616587)	*Oligotropha carboxidovorans* OM5 (CP001196)	90	*Oligotropha carboxidovorans* OM5 (CP001196)	90	59.7	67.2
	2 (HE616588)	*Salinispora arenicola* CNS-205 (CP000850)	77	*Salinispora arenicola* CNS-205 (CP000850)	77	33.4	9.0

	3 (HE616589)	uncultured bacterium (FJ556669)	88	*Marinithermus hydrothermalis* DSM 14884 (CP002630)	75	6.4	23.7

*nirK*	1 (HE616593)	uncultured bacterium (GU270516)	86	*Brucella canis* ATCC 23365 (NC 010104)	82	30.4	71.9

	2 (HE616594)	uncultured bacterium (DQ784043)	96	*Rhizobium etli* CFN 42 (NC 007766)	95	61.7	2.6

	3 (HE616595)	*Castellaniella* sp. ROi28 (EF363542)	86	*Castellaniella* sp. ROi28 (EF363542)	86	0.1	17.1

	4 (HE616596)	*Alcaligenes* sp. ESPY2 (EF202174)	80	*Alcaligenes* sp. ESPY2 (EF202174)	80	0.0	3.9

	5 (HE616597)	*Bosea* sp. MF18 (EF363545)	100	*Bosea* sp. MF18 (EF363545)	100	0.9	2.3

	6 (HE616598)	Ochrobactrum intermedium *LMG 3301 (NZ ACQA01000001)*	91	Ochrobactrum intermedium *LMG 3301 (NZ ACQA01000001)*	91	1.1	0.7

	7 (HE616599)	uncultured bacterium (FJ204551)	86	*Sinorhizobium* sp. R-24605 (AM230817)	78	1.3	0.1

	8 (HE616600)	uncultured bacterium (FJ204565)	88	*Afipia* sp. 4AS1 (GQ404514)	75	1.0	0.2

*nirS*	1 (HE616602)	uncultured bacterium (AY583422)	90	*Thiobacillus denitrificans* ATCC 25259 (CP000116)	74	51.9	6.2

	2 (HE616603)	uncultured bacterium (GU393229)	88	*Rhodanobacter* sp. D206a (AB480490)	84	9.0	25.7

	3 (HE616604)	uncultured bacterium (GU393200)	88	*Thiobacillus denitrificans* ATCC 25259 (CP000116)	72	1.5	25.1

	4 (HE616605)	uncultured bacterium (DQ676073)	88	*Aromatoleum aromaticum* EbN1 (NC 006513)	74	25.3	1.7

	5 (HE616606)	uncultured bacterium (DQ676123)	86	*Thiobacillus denitrificans* ATCC 25259 (CP000116)	77	1.0	16.7

	6 (HE616607)	uncultured bacterium (GU393132)	84	Cupriavidus sp. N75 (AB480489)	79	0.3	12.7

	7 (HE616608)	uncultured bacterium (GU393183)	86	*Sideroxydans lithotrophicus* ES-1 (CP001965)	81	0.03	6.3

	8 (HE616609)	uncultured bacterium (HM438800)	95	*Cupriavidus metallidurans* CH34 (CP000352)	77	2.4	3.1

	9 (HE616610)	uncultured bacterium (DQ676073)	91	*Dechloromonas* sp. R-28400 (AM230913)	77	2.4	0. 3

	10 (HE616611)	uncultured bacterium (GQ443982)	87	*Aromatoleum aromaticum* EbN1 (NC 006513)	81	2.4	0.2

	11 (HE616612)	uncultured bacterium (AM419582)	94	*Arthrobacter* sp. TSA68 (AB542303)	80	2.1	0.2

*nosZ*	1 (HE616616)	uncultured bacterium (DQ010777)	99	*Bosea* sp. PD 24 (DQ377796)	89	60.7	88.3

	2 (HE616617)	*Achromobacter* sp. PD 27 (DQ377799)	85	*Achromobacter* sp. PD 27 (DQ377799)	85	13.6	5.3

	3 (HE616618)	*Azospirillum largimobile* ACM 2041 (AY072228)	88	*Azospirillum largimobile* ACM 2041 (AY072228)	88	13.1	0.6

	4 (HE616619)	uncultured bacterium (FN859926)	95	*Herbaspirillum* sp. TSA29 (AB542280)	75	7.3	1.5

	5 (HE616620)	uncultured bacterium (FN859707)	98	*Ralstonia solanacearum* GMI1000 (AL646053)	73	3.3	1.5

	6 (HE616621)	uncultured bacterium (DQ324384)	90	*Ralstonia eutropha* H16 (NC 005241)	73	0.5	1.3

	7 (HE616622)	uncultured bacterium (FN430515)	99	*Rhodobacter sphaeroides* f. sp. *denitrificans* IL106 (AF125260)	78	0.0	1.3
* *							

^1^ Determined after alignment in MEGA 5.0.

*nirK* were assigned to 24 species-level OTUs in total. 23 and 17 OTUs were detected in fen soil from 0 to 20 cm and from 20 to 40 cm, respectively (Table 3). Community composition differed significantly between the soil layers (Fig 4 B). OTU 2 dominated *nirK* in fen soil from 0 to 20 cm (about 60%), while OTU 1 dominated *nirK* in fen soil from 20 to 40 cm, respectively (about 70%; Fig 4 B). Similarities of OTU representative sequences to *nirK* of cultured organisms ranged from 75–100% (Table 4). Most OTUs were related to *Alphaproteobacterial nirK*. OTUs 1, 2, and 3 were related to *nirK* of *Brucella canis*, *Rhizobium etli*, and *Castellaniella* sp., respectively (Table 4 and S4 Fig). Further OTUs were related to *nirK* of *Bosea* sp., *Afipia* sp., or uncultured bacteria (Table 4 and S4 Fig). *nirS* were assigned to 25 species-level OTUs in total. 22 and 23 OTUs were detected in fen soil from 0 to 20 cm and from 20 to 40 cm, respectively (Table 3). Differences in community composition of *nirS* from the soil layers were more pronounced than those of *nirK* (Fig 4 B and 4 C). *nirS* of fen soil was dominated by OTUs affiliated to *Beta-* and *Gammaproteobacterial nirS*. However, about 26% of detected *nirS* from 20 to 40 cm affiliated with *Alphaproteobacterial nirS* (S5 Fig). *nirS* of OTU representatives were only distantly related to *nirS* of cultured organisms (i.e., similarities ranged from 74–84%, Table 4). Many OTUs of both soil layers were related to *nirS* of uncultured wetland or marine sediment bacteria, and distantly related to *nirS* of e.g., *Thiobacillus denitrificans*, *Dechloromonas* sp., and *Arthrobacter* sp. (Table 4 and S5 Fig). Diversity estimates calculated from rarified OTU tables of *nirK* based on species-level threshold similarities differed significantly between 0–20 and 20–40 cm fen soil (Table 3). Chao1 richness estimates of *nirS* did not differ significantly at species-level similarity thresholds, amounting to about 24 in both soil layers, while Shannon diversity as well as species evenness were significantly higher in the lower soil layer (Table 3). On the contrary, Shannon diversity, species Evenness, and Chao1 richness estimates of *nirK* and *nirS* calculated from rarified OTU tables based on 97% threshold similarity were consistently higher in 0 to 20 cm than 20–40 cm fen soil (Table 3).

*nosZ* forward reads were assigned to 10 species-level OTUs in total. 8 OTUs were detected in each soil layer (Table 3). OTU 1 dominated *nosZ* of fen soil from both soil layers (Fig 4 D). Essentially all *nosZ* from both soil layers affiliated with *Alpha*- and *Betaproteobacterial nosZ* (S6 Fig). Most *nosZ* sequences from fen soil were distantly related to *nosZ* of cultured organisms with sequence dissimilarities ranging from 11–27% (Table 4), indicating hitherto uncultured denitrifiers capable of N_2_O reduction in fen soil. *nosZ* sequences clustered with *nosZ* of wetland and upland soils, as well as *Achromobacter* sp., *Herbaspirillum* sp., and *Ralstonia* sp. (Table 4 and S6 Fig). Shannon diversity and species evenness calculated from rarified OTU tables at species-level threshold similarity were significantly higher in 0 to 20 cm than in 20 to 40 cm soil, while there was no significant difference in Chao1 richness estimates (Table 3). At 97% threshold similarity, all diversity estimates calculated from rarified OTU tables were significantly higher in the upper soil layer (Table 3). The difference in threshold similarity most strongly affected on the number of observed and estimated OTUs, which were similar at species-level threshold similarity (around 8 in both soil layers), but were about 3 times higher in 0 to 20 cm soil at 97% similarity threshold (Table 3). Beta-diversity was higher at 97% than at species-level threshold similarity (Table 3).

### Quantification of *narG*, *nirK*, *nirS*, and *nosZ* relative to 16S rRNA genes

**Fig 5:**
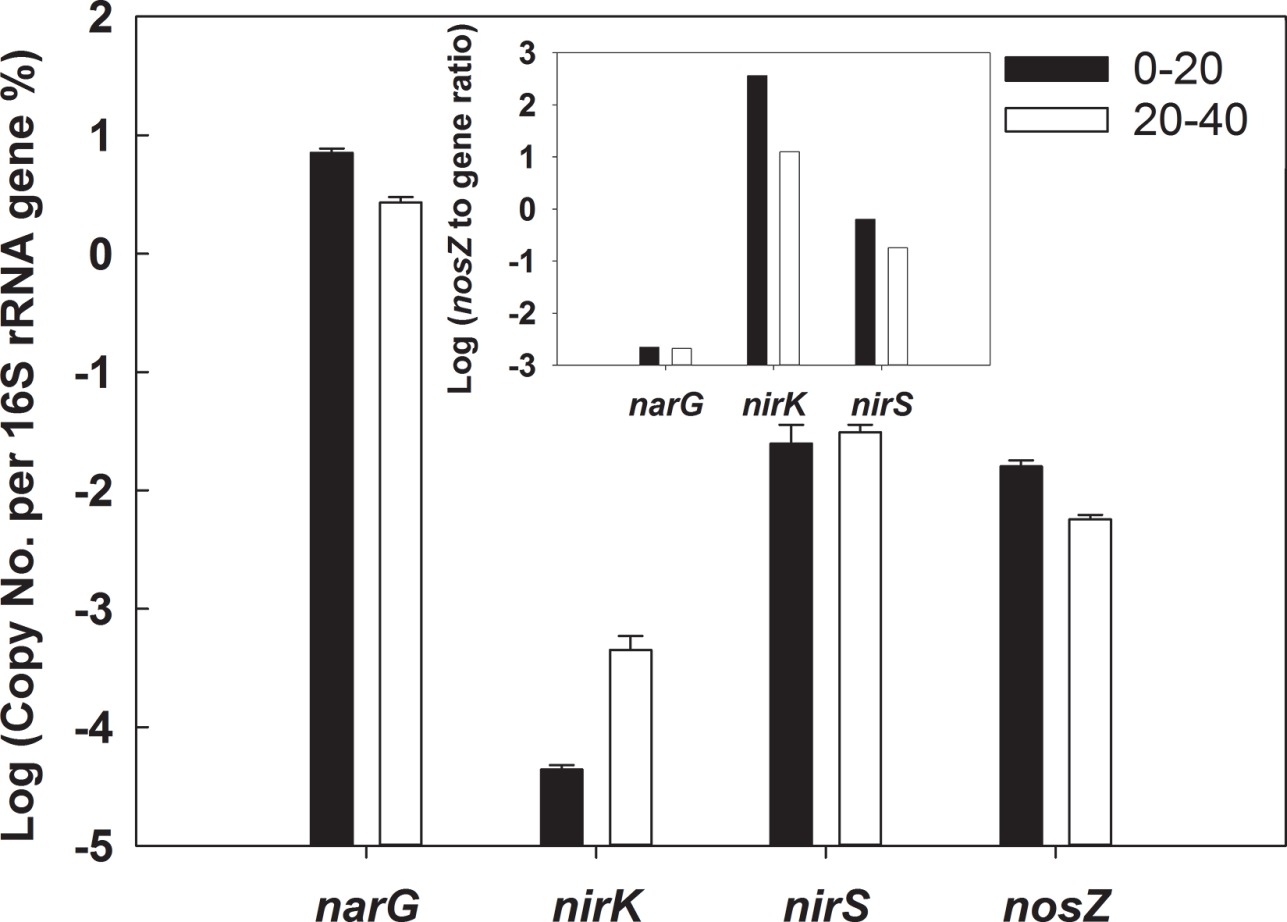
Abundance of denitrification associated genes normalized by 16S rRNA gene copy numbers in pH-neutral fen soil. The inset shows ratios of normalized *nosZ* gene abundances and *narG*, *nirK*, as well as *nirS*. Mean values and standard errors of six replicates are displayed. Black and white bars indicate fen soil from 0–20 and 20–40 cm depth, respectively.

Copy numbers of all genes investigated in this study were corrected by inhibition factors that were experimentally determined for every DNA extract and gene analyzed (see Material and Methods). 16S rRNA gene copy numbers of 0–20 and 20–40 cm fen soil were (5.3 ± 0.3) x 10^5^ and (8.6 ± 0.3) x 10^5^ per ng DNA. Copy numbers of *narG* accounted for 7 and 3% of bacterial 16S rRNA gene copy numbers in 0 to 20 cm and 20 to 40 cm fen soil, respectively (Fig 5). Copy numbers of *nirK*, *nirS*, and *nosZ* were lower than *narG* copy numbers (Fig 5). Copy numbers of *nirS* were app. 100x and 10x higher than copy numbers of *nirK* and *nosZ*, respectively, in both soil layers (Fig 5). Copy numbers of *narG* and *nosZ* were 3 x higher (*P* < 0.01), and those of *nirK* were slightly lower in 0 to 20 cm than 20 to 40 cm fen soil (*P* = 0.1). Ratios of *nosZ* to *narG* were similar in both soil layers. Those of *nosZ* to *nirK* and *nirS* were 30 and 3 x higher, respectively, in 0 to 20 cm than 20 to 40 cm fen soil (Fig 5).

## Discussion

### pH neutral fen soil as N_2_O sink

Peatlands are important ecosystems in the northern hemisphere and cover more than 30% of the Finnish land surface [36]. The potential of those peatlands to produce or consume greenhouse gases is of great interest, especially in respect to climate warming which is predicted to have a strong impact on peatlands [37]. N_2_O emissions from natural wetlands are highly variable, and many water-saturated soils are also sinks for N_2_O [2, 16, 17]. Many studies demonstrate that undrained, pH-neutral and acidic fens are sources of molecular nitrogen, and act as sinks for N_2_O depending on environmental conditions [10, 14, 16, 38–40]. N_2_O accumulation in gas chamber experiments from Puukkosuo fen were also variable, ranging from 10 ppb to -17 ppb at the time of soil sampling. Fen soil *in situ* consumed initially produced N_2_O during nitrate or ammonium fertilization experiments (Fig 1). Previous studies show that mainly complete denitrification to N_2_ occurs in pristine pH-neutral fens at *in situ* nitrate concentrations [10, 40]. Thus, the absence of *in situ* N_2_O emission from Puukkosuo fen soil is likely due to complete denitrification to N_2_ as the major end product (Fig 1). Even though the amount of stored nitrogen in the soil is high, low concentrations of available nitrate are observed in a northern boreal fen, where denitrification is thus N-limited [10]. Nitrate concentrations in Puukkosuo fen soil were likewise low, nitrite was not detected (Table 1), and nitrate as well as nitrite stimulated denitrification (Fig 3), indicating nitrate- and nitrite-limitation of fen denitrifiers. Microcosms and *in situ* fertilization with nitrate resulting in temporary *in situ* emission of N_2_O with subsequent consumption indicated ongoing complete denitrification (Fig 1).

Peatland soils are temporarily or permanently water-logged, and oxygen generally penetrates only the uppermost centimeters, leading to oxygen-limitation in lower soil layers. In the absence of oxygen and nitrate, nitrous oxide is a potent sink for electrons released during the oxidation of organic carbon compounds, as the reduction of N_2_O by H_2_ is even more exergonic than O_2_ reduction by H_2_ (N_2_O half-cell potential of *E*_*0*_^’^ (pH 7.0) = 1.35 V; *ΔG*_*0*_^’^ *=* -339.5 kJ*mol^-1^; reviewed in [41]). *In situ* relevant concentrations of dissolved organic carbon (app. 5 μM glucose equivalents; e.g., [42]) and atmospheric concentrations of N_2_O result in -360 kJ*mol_N2O_^-1^ (http://cms.uni-konstanz.de/schink/dg-calculator/), allowing for high energy conservation of organisms capable of N_2_O reduction. Lower below-surface N_2_O concentrations than at atmospheric equilibrium are observed in fens and suggest ongoing N_2_O consumption [10, 16, 38]. Indeed, apparent *K*_*M*_ values of Puukkosuo fen soil were approximately 60 times lower for N_2_O than for nitrate (Table 2), indicating a higher affinity of fen denitrifiers for N_2_O than for nitrate. The assumed absence of oxygen, the observed nitrate-limitation and high N_2_O affinity indicate a strong *in situ* sink potential of Puukkosuo fen for N_2_O.

### Diverse denitrifier communities are associated with denitrification activities in pH-neutral fen soil

Unsupplemented fen soil from both sampled soil layers produced N_2_O in acetylene-amended microcosms, demonstrating the denitrification potential of the fen soil. However, nearly no N_2_O was produced in the absence of acetylene and initially produced N_2_O was subsequently consumed (Fig 2). Nitrate- and oxygen-limitation might select for denitrifiers capable of complete denitrification, and hitherto unknown denitrifiers as well as N_2_O reducers might occur in Puukkosuo fen soil. Indeed, *nosZ* copy numbers in 0 to 20 cm fen soil were of a similar magnitude as nitrite reductase copy numbers (Fig 5), and newly-discovered *nirK/S* and *nosZ* (Table 4) indicate that a high percentage of uncharacterized denitrifiers in that soil layer possessed a complete denitrification pathway.

Supplemental nitrate and nitrite resulted in immediate N_2_O production in fen soil after internal nitrate and nitrite were consumed. Stimulation was greater with nitrite than with nitrate in both soil layers (Fig 3 A). This reflects the fact that all denitrifiers “sensu stricto” use nitrite as electron acceptor, while many cultured denitrifiers lack the ability to use nitrate as electron acceptor [20, 43]. Stimulation was also greater in the top soil layer (Fig 3 A), reflecting a greater denitrification potential of the top soil. In other wetland and also agricultural soils, denitrification potentials are also highest in the top soil layers (e.g., [14, 44]). In 20 to 40 cm fen soil, N_2_O production and total denitrification decreased with increasing nitrate concentrations, indicating substrate inhibition of denitrification at high nitrate concentrations. This finding is in contrast to denitrification potentials reported for deeper horiozonts of agricultural soils, suggesting that the fen denitrifier community of 20–40 cm depth is well adapted to low nitrate concentrations (e.g., [44]). Nitrate and nitrite reduction compete for electrons at high nitrate concentrations, and nitrate reduction is favored over the rest of the denitrification pathway, causing eventually accumulation of nitrite when electron donors are limiting [45].

The ratio of N_2_O to (N_2_+N_2_O) was lower in nitrate- and nitrite-amended microcosms with 0 to 20 cm fen soil than in 20 to 40 cm fen soil when nitrate or nitrite were supplied (S2 Fig), and consumption of supplied N_2_O was about 2-fold higher in 0 to 20 cm than in 20 to 40 cm fen soil (Fig 3 B). Indeed, the ratio of nitrite to nitrous oxide reductases was higher in 20 to 40 cm fen soil than in 0 to 20 cm fen soil (Fig 5), indicating an increased amount of denitrifiers lacking nitrous oxide reductase in the lower soil layer. The ratio of nitrite reductase genes to N_2_O reductase genes is highly variable in soils, and often nitrite reductase copy numbers largely exceed N_2_O reductase copy numbers [15, 23, 46]. However, non-denitrifying N_2_O consumers were recently shown to be quantitatively important in certain soils [47, 48]. Relative abundances of both atypical and typical *nosZ* assigned to non-denitrifiers and denitrifiers, respectively, are variable in soil metagenomes. Hence, further analyses including both groups are demanded for better understanding of N_2_O reducers in fens [49]. Nevertheless, N_2_O produced in lower layers of fen soil can diffuse upwards and be further reduced to N_2_ in upper soil layers, and thus emission of N_2_O into the atmosphere can be reduced [14, 16, 38]. It is thus hypothesized that also in Puukkosuo fen soil lower soil layers are N_2_O sources while upper soil layers are N_2_O sinks.

The analysis of denitrification-specific gene markers indicated a higher diversity of these genes in 0 to 20 cm than in 20 to 40 cm fen soil (Table 3). Detected *narG* and *nosZ* were more similar in 0 to 20 cm and 20 to 40 cm fen soil than *nirK* and *nirS* (Fig 4), indicating that nitrite reductases show a higher variability in fen soil than nitrate and N_2_O reductases. Nitrite reductase community composition is highly variable in other types of peatland soils, including permafrost affected systems, while variations in nitrate and N_2_O reductase community composition are much less pronounced [15, 23]. Indeed, the distribution of nitrite reductases is more heavily impacted by changes in environmental conditions than those of nitrate or N_2_O reductases [50–52]. Nitrite reductase genes from fen soil were affiliated with *Proteobacterial nirK/S* (Table 4 and S4 Fig and S5 Fig). For *nirS*, sequences related to *Rhodanobacter/Bradyrhizobium* were detected (S5 Fig). Such sequences are also detected in other peatland soils such as permafrost affected tundra and palsa peat soils [15, 23]. *Proteobacteria*-affiliated sequences of *narG* and *nosZ* (Table 4 and S3 Fig and S6 Fig) further support that *Proteobacteria* play an important role for denitrification in this pH-neutral fen soil. Denitrification-associated genes related to *Proteobacteria* are also found in acidic fen soils or permafrost-affected peatlands [14, 15, 23], indicating that *Proteobacteria* represent general peatland denitrifiers. Sequences of *narG* were also affiliated with *Actinobacterial narG* (10–30%; S3 Fig). *Actinobacteria* are common in soils, include many genera capable of nitrate reduction, and are in general considered to be more tolerant to extreme environmental conditions such as low pH or low temperature [53–55]. *Actinobacteria* and *Actinobacteria*-affiliated gene markers are frequently detected in a variety of peatlands including acidic fen soils, permafrost-affected tundra and palsa peat soils [14, 15, 23]. However, in those more extreme environments, *Actinobacteria* often dominate the *narG* communities, indicating that *Actinobacteria* are further important players involved in nitrate reduction and potentially denitrification in pH-neutral fen soil [15, 23]. The nitrate reducer community in pH-neutral fen soil also contained a substantial portion of *Deinococci*-affiliated *narG* (Fig 4A and S3 Fig.), which are not detected in the above mentioned more extreme habitats such as acidic fens, frost-affected tundra and palsa peat soils [15, 23]. Soil pH is a driver of the general microbial community structure [56]. Denitrifier diversity in pH-neutral fen soil is high when compared to more acidic pristine peatland soils [14, 15, 23], suggesting that soil pH likewise plays an important role in shaping denitrifier communities.

Denitrifier diversity and quantity is routinely underestimated due to choice of primer sets, e.g., gram-positive denitrifiers escaped detection in many studies [46, 50, 57]. Soil metagenomes might represent an alternative strategy to obtain a more complete picture of denitrifier diversity in soils. However, the low abundance of denitrification associated genes on denitrifier genomes (i.e., app. 1%; most of the genes on denitrifier genomes are associated with other functions than denitrification like anabolism, motility, etc.) in combination with a low number of denitrifiers compared to total number of prokaryotes in soil (app. 1%) limits their detection by metagenomics [49, 58, 59]. However, metagenomes are extremely useful for the design of denitrification gene specific primers. Although amplicon based approaches combined with next generation sequencing depend on the choice of primers, such approaches currently provide a cost-effective way for the detection of a large denitrifier diversity.

The collective data indicate that (i) a core nitrate reducer/denitrifier community might be common to all kinds of (northern) peatlands, (ii) some nitrate reducers/denitrifiers are unique in pH-neutral fen soil, possible due to the lack of environmental stress that might be induced by acidic pH, (iii) denitrifier communities are from upper and lower layers are dissimilar as indicated by apparent Michaelis-Menten kinetics and structural gene marker analyses, and (iv) pH-neutral fens are a strong potential sink for atmospheric N_2_O.

## Supporting Information

S1 Fig Effect of supplemental nitrate (1) and nitrite (2) on N_2_O production and consumption in microcosms with fen soil. Squares and circles represent fen soil from 0 to 20 cm and 20 to 40 cm depth, respectively. Microcosms with and without acetylene are represented by closed and open symbols, respectively. Supplied concentrations of nitrate or nitrite were 0 μM (A), 10 μM (B), 20 μM (C), 50 μM (D), 100 μM (E), 500 μM (F), and 1000 μM (G). Mean values and standard errors of three replicate microcosms are shown.
(TIF)

S2 Fig Effect of supplemental nitrate (black) or nitrite (white) on the ratio of N_2_O to (N_2_+N_2_O) in anoxic microcosms with fen soil from 0 to 20 cm (A) and 20 to 40 cm (B) depth. Mean values and standard errors of three replicates are shown.
(TIF)

S3 Fig Phylogenetic tree of *narG* OTU representatives detected in 0 to 20 cm and 20 to 40 cm fen soil. The trees was calculated based in translated amino acid sequences of *narG* forward reads. OTUs were grouped at a species-level threshold dissimilarity of 33%. Numbers preceeding sequence names refer to sequence accession numbers of reference sequences from public databases. Values given in parentheses show the relative abundances of each OTU in 0 to 20 cm (left) and 20 to 40 cm (right) fen soil. Grey boxes indicate reference sequences belonging to the same phylogenetic group. The percentage of replicate trees that produced the observed clustering of taxa in the bootstrap test (10 000 replications) are shown next to the branches. Bootstrap supports below 50% are not displayed. *narG* of *Haloarcula marismortui* ATCC 43049 was used as outgroup to root the tree.
(TIF)

S4 Fig Phylogenetic tree of *nirK* OTU representatives detected in 0 to 20 cm and 20 to 40 cm fen soil. The tree was calculated based on translated amino acid sequences. OTUs were grouped at a species-level threshold dissimilarity of 17%. Numbers preceeding sequence names refer to sequence accession numbers of reference sequences from public databases. Values given in parentheses show the relative abundances of each OTU in 0 to 20 cm (left) and 20 to 40 cm (right) fen soil. Grey boxes indicate reference sequences belonging to the same phylogenetic group. The percentages of replicate trees that produced the observed clustering of taxa in the bootstrap test (10 000 replications) are shown next to the branches. Bootstrap supports below 50% are not displayed. *nirK* of *Nitrosomonas* sp. C-56 was used as outgroup to root the tree.
(TIF)

S5 Fig Phylogenetic tree of *nirS* OTU representatives detected in 0 to 20 cm and 20 to 40 cm fen soil. The tree was calculated based on translated amino acid sequences. OTUs were grouped at a species-level threshold dissimilarity of 18%. Numbers preceeding sequence names refer to sequence accession numbers of reference sequences from public databases. Values given in parentheses show the relative abundances of each OTU in 0 to 20 cm (left) and 20 to 40 cm (right) fen soil. Grey boxes indicate reference sequences belonging to the same phylogenetic group, white boxes indicate single taxa not belonging to the major phylogenetic group. The percentages of replicate trees that produced the observed clustering of taxa in the bootstrap test (10 000 replications) are shown next to the branches. Bootstrap supports below 50% are not displayed. *nirS* of *Rhodothermus marinus* DSM 4252 was used as outgroup to root the tree.
(TIF)

S6 Fig Phylogenetic tree of *nosZ* OTU representatives detected in 0 to 20 cm and 20 to 40 cm fen soil. The tree was calculated based on translated amino acid sequences of *nosZ* forward reads. OTUs were grouped at a species-level threshold dissimilarity of 20%. Numbers preceeding sequence names refer to sequence accession numbers of reference sequences from public databases. Values given in parentheses show the relative abundances of each OTU in 0 to 20 cm (left) and 20 to 40 cm (right) fen soil. Grey boxes indicate reference sequences belonging to the same phylogenetic group, white boxes indicate single taxa not belonging to the major phylogenetic group. The percentages of replicate trees that produced the observed clustering of taxa in the bootstrap test (10 000 replications) are shown next to the branches. Bootstrap supports below 50% are not displayed. *nosZ* of *Haloarcula marismortui* ATCC 43049 was used as outgroup to root the tree.
(TIF)
