# Supplementary figures and images for: Denitrification Activity of a Remarkably Diverse Fen Denitrifier Community in Finnish Lapland Is N-Oxide Limited
Source: PLoS One. 2015 Apr 10;10(4):e0123123. doi: 10.1371/journal.pone.0123123 (PMC4393310; doi:10.1371/journal.pone.0123123)

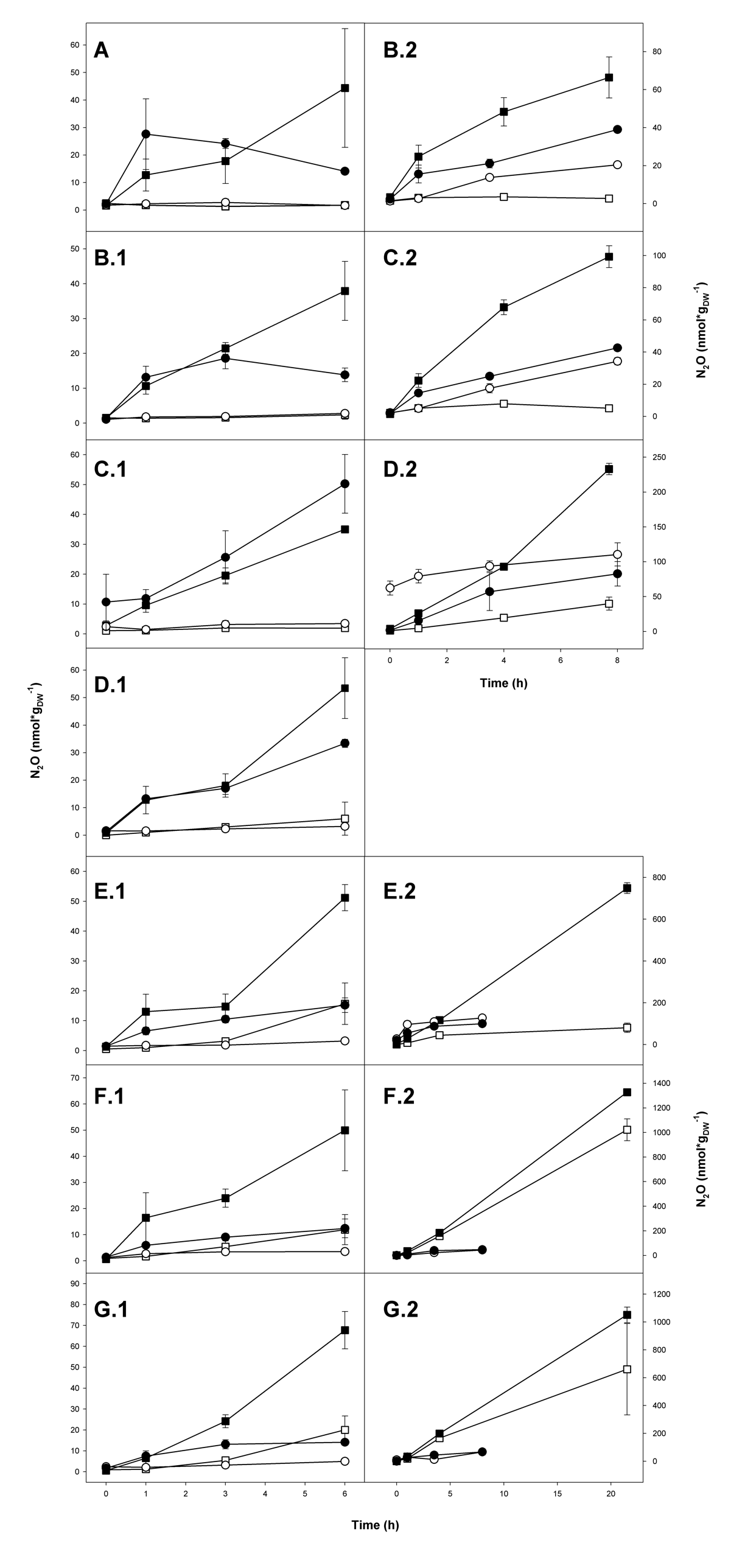

Supplement: S1 Fig — Squares and circles represent fen soil from 0 to 20 cm and 20 to 40 cm depth, respectively. Microcosms with and without acetylene are represented by closed and open symbols, respectively. Supplied concentrations of nitrate or nitrite were 0 μM (A), 10 μM (B), 20 μM (C), 50 μM (D), 100 μM (E), 500 μM (F), and 1000 μM (G). Mean values and standard errors of three replicate microcosms are shown. (TIF) [file pone.0123123.s001.tif]

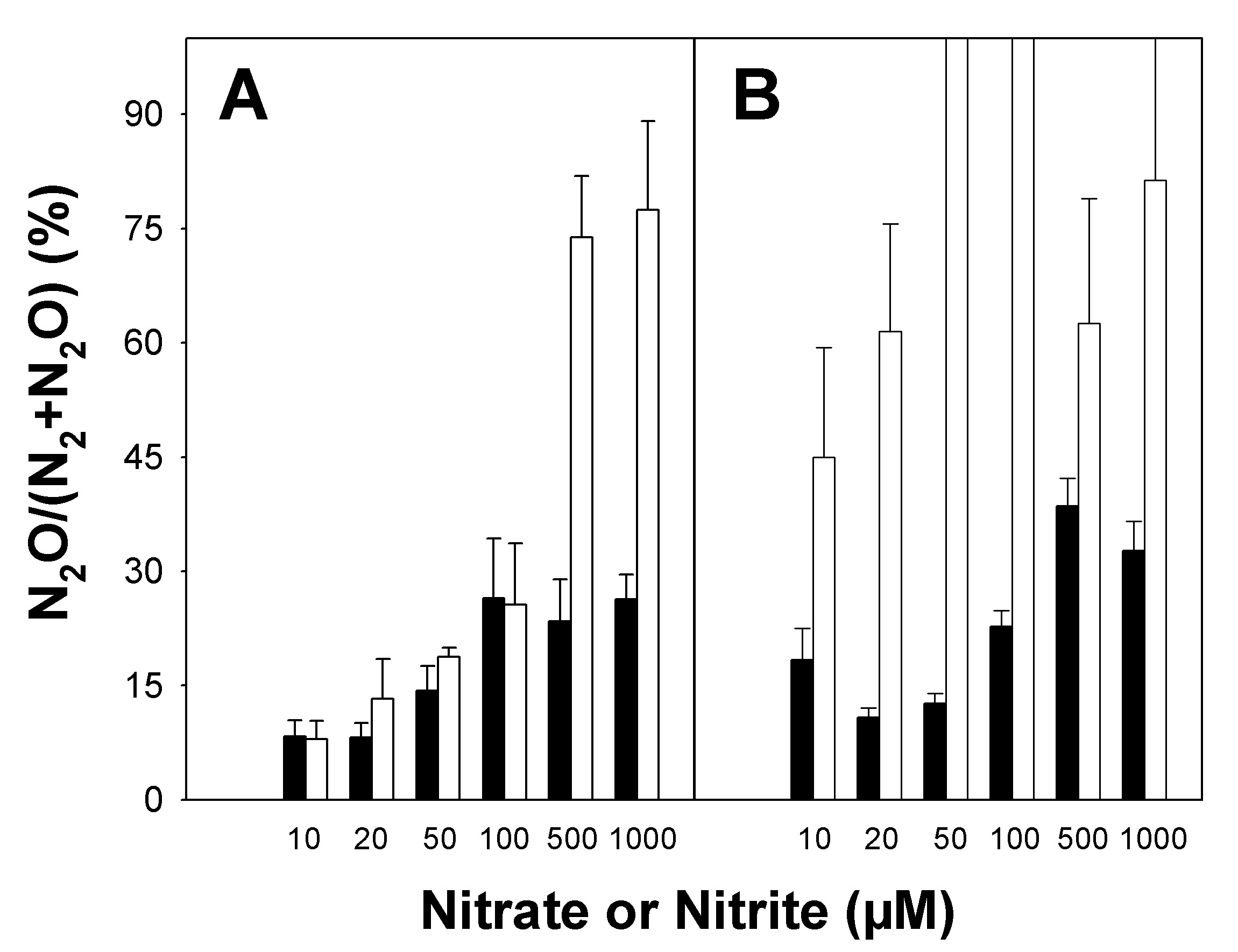

Supplement: S2 Fig — Mean values and standard errors of three replicates are shown. (TIF) [file pone.0123123.s002.tif]

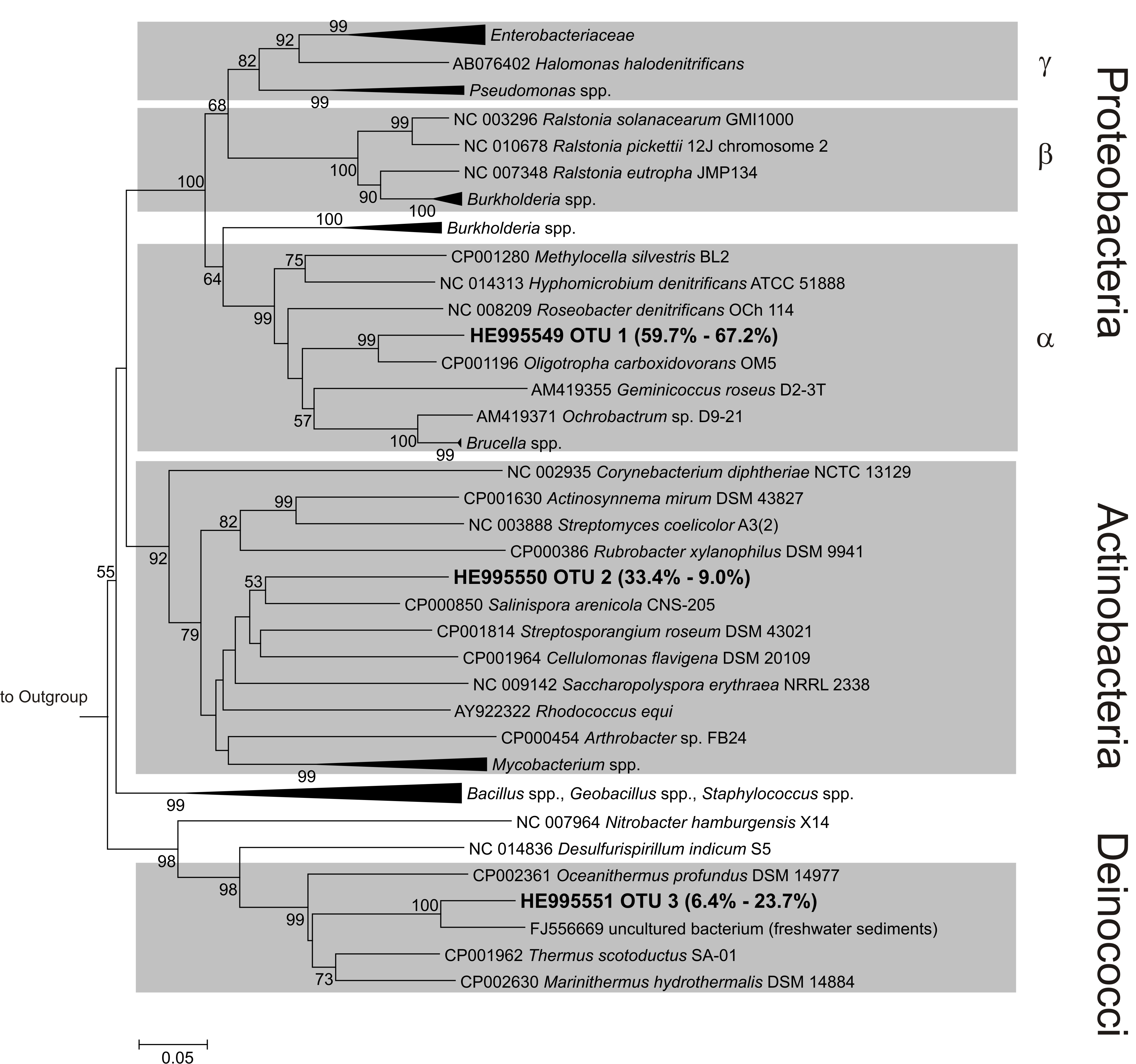

Supplement: S3 Fig — The trees was calculated based in translated amino acid sequences of narG forward reads. OTUs were grouped at a species-level threshold dissimilarity of 33%. Numbers preceeding sequence names refer to sequence accession numbers of reference sequences from public databases. Values given in parentheses show the relative abundances of each OTU in 0 to 20 cm (left) and 20 to 40 cm (right) fen soil. Grey boxes indicate reference sequences belonging to the same phylogenetic group. The percentage of replicate trees that produced the observed clustering of taxa in the bootstrap test (10 000 replications) are shown next to the branches. Bootstrap supports below 50% are not displayed. narG of Haloarcula marismortui ATCC 43049 was used as outgroup to root the tree. (TIF) [file pone.0123123.s003.tif]

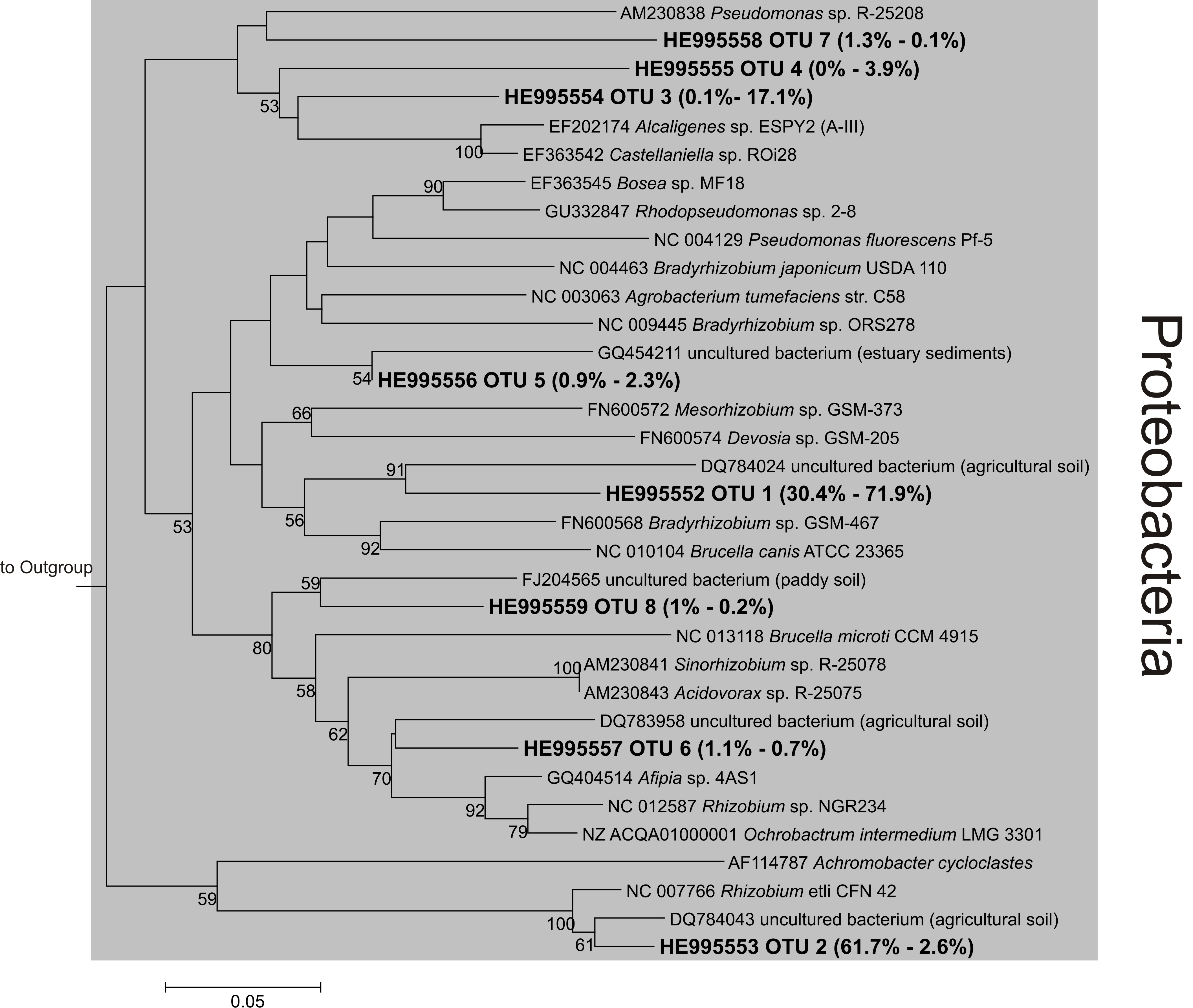

Supplement: S4 Fig — The tree was calculated based on translated amino acid sequences. OTUs were grouped at a species-level threshold dissimilarity of 17%. Numbers preceeding sequence names refer to sequence accession numbers of reference sequences from public databases. Values given in parentheses show the relative abundances of each OTU in 0 to 20 cm (left) and 20 to 40 cm (right) fen soil. Grey boxes indicate reference sequences belonging to the same phylogenetic group. The percentages of replicate trees that produced the observed clustering of taxa in the bootstrap test (10 000 replications) are shown next to the branches. Bootstrap supports below 50% are not displayed. nirK of Nitrosomonas sp. C-56 was used as outgroup to root the tree. (TIF) [file pone.0123123.s004.tif]

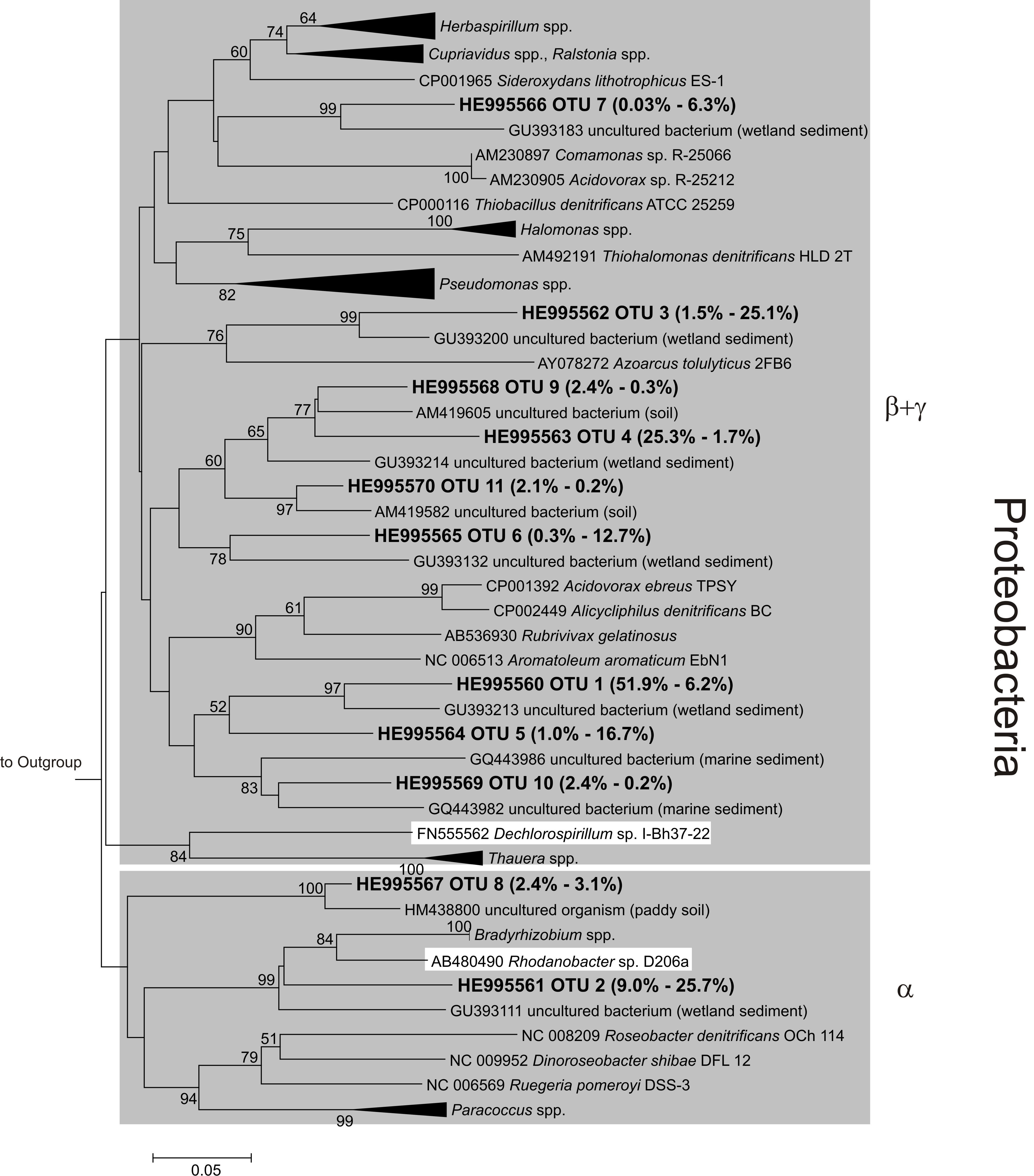

Supplement: S5 Fig — The tree was calculated based on translated amino acid sequences. OTUs were grouped at a species-level threshold dissimilarity of 18%. Numbers preceeding sequence names refer to sequence accession numbers of reference sequences from public databases. Values given in parentheses show the relative abundances of each OTU in 0 to 20 cm (left) and 20 to 40 cm (right) fen soil. Grey boxes indicate reference sequences belonging to the same phylogenetic group, white boxes indicate single taxa not belonging to the major phylogenetic group. The percentages of replicate trees that produced the observed clustering of taxa in the bootstrap test (10 000 replications) are shown next to the branches. Bootstrap supports below 50% are not displayed. nirS of Rhodothermus marinus DSM 4252 was used as outgroup to root the tree. (TIF) [file pone.0123123.s005.tif]

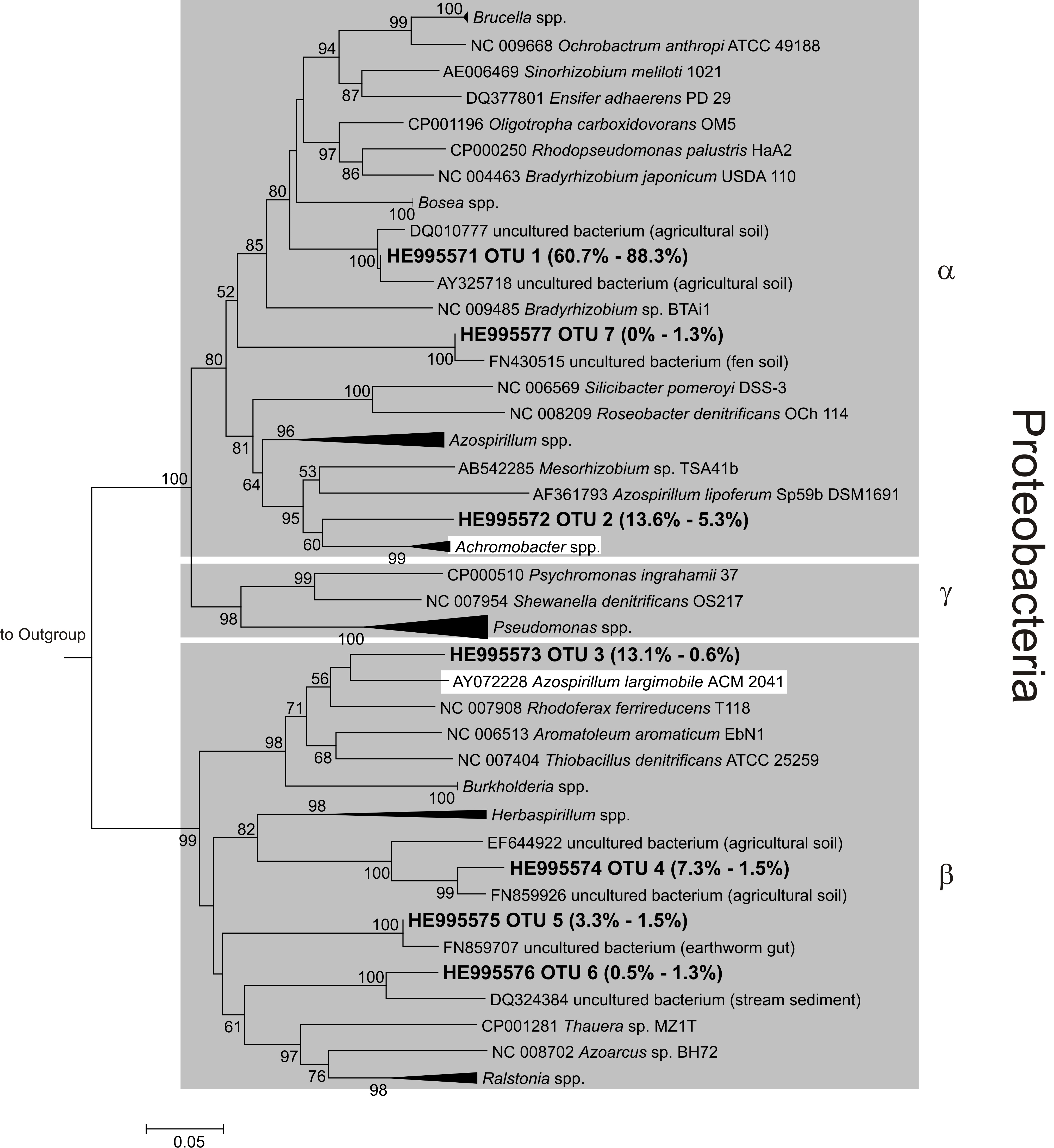

Supplement: S6 Fig — The tree was calculated based on translated amino acid sequences of nosZ forward reads. OTUs were grouped at a species-level threshold dissimilarity of 20%. Numbers preceeding sequence names refer to sequence accession numbers of reference sequences from public databases. Values given in parentheses show the relative abundances of each OTU in 0 to 20 cm (left) and 20 to 40 cm (right) fen soil. Grey boxes indicate reference sequences belonging to the same phylogenetic group, white boxes indicate single taxa not belonging to the major phylogenetic group. The percentages of replicate trees that produced the observed clustering of taxa in the bootstrap test (10 000 replications) are shown next to the branches. Bootstrap supports below 50% are not displayed. nosZ of Haloarcula marismortui ATCC 43049 was used as outgroup to root the tree. (TIF) [file pone.0123123.s006.tif]
